# Lithium and coronaviral infections. A scoping review.

**DOI:** 10.12688/f1000research.22299.2

**Published:** 2020-04-03

**Authors:** Jan K. Nowak, Jarosław Walkowiak

**Affiliations:** 1 Department of Pediatric Gastroenterology and Metabolic Diseases, Poznan University of Medical Sciences, Poznan, Poland

**Keywords:** coronavirus, Coronaviridae, Wuhan, 2019-nCoV, lithium, lithium carbonate, lithium orotate, antiviral, apoptosis, glycogen synthase kinase 3-beta, GSK-3β

## Abstract

The current rapid spread of the novel coronavirus (SARS-CoV-2) causing coronavirus disease 2019 (COVID-19) calls for a rapid response from the research community. Lithium is widely used to treat bipolar disorder, but has been shown to exhibit antiviral activity. This brief review took a systematic approach to identify six *in vitro* studies reporting on the influence of lithium on coronaviral infections. We propose mechanistic investigation of the influence of lithium – alone and with chloroquine – on the SARS-CoV-2 infection.

## Introduction

The current rapid spread of severe acute respiratory syndrome coronavirus 2 (SARS-CoV-2) causing coronavirus disease 2019 (COVID-19), calls for a rapid response from the research community. Lithium is known to exhibit antiviral activity, but the knowledge of its potential as a possible therapy for coronoviral infections has not been summarized yet. The aim of this brief report is to draw attention to lithium as potential COVID-19 treatment and prophylaxis.

## Methods

On February 1 ^st^ 2020 the following PubMed search was conducted with no language or time restrictions: (lithium and (coronavirus or *coronavirus or sarbecovirus or SARS or “severe acute respiratory syndrome” or MERS or “Middle East respiratory syndrome” or nobecovirus or merbecovirus or hibecovirus or embecovirus or andecovirus or buldecovirus or herdecovirus or moordecovirus or cegacovirus or igacovirus or “microhyla lentovirus” or milecovirus or alphaletovirus or tegacovirus or setracovirus or rhinacovirus or pedacovirus or “porcine epidemic diarrhea” or nyctacovirus or “nectalus velutinus” or myotacovirus or “myotis ricketti” or minunacovirus or minacovirus or luchacovirus or duvinacovirus or decacovirus or “Rhinolophus ferrumequinum” or “transmissible gastroenteritis virus” or “feline infectious peritonitis virus” or “canine coronavirus” or “murine hepatitis virus”)). The search yielded 45 articles, of which all the abstracts were charted and reviewed by two researchers.

## Results

**Figure 1.**
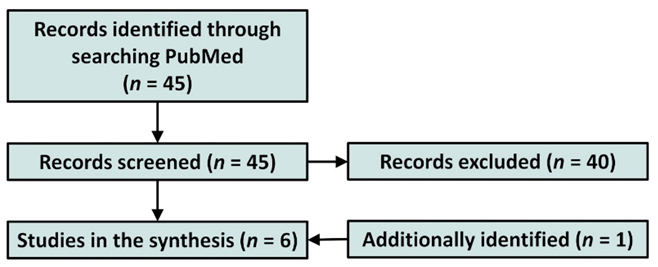
Study flow chart.

Six studies reporting on the influence of lithium on coronaviral infections were identified ( Figure 1).

In Vero cells, lithium chloride (investigated at 1–15 mM) was shown to be dose-dependently effective in suppressing infection with the porcine epidemic diarrhea virus (PEDV), a member of the Coronaviridae family ^1^. Not only PEDV entry and replication were inhibited in the presence of LiCl, but apoptosis as well. Yet, LiCl at 1 mM (safe in patients) was not effective. At 5 mM LiCl reduced viral RNA levels by 30% (p < 0.001). In MARC-145 cells, LiCl reduced the production of RNA and proteins specific to the porcine reproductive and respiratory syndrome virus. The relative viral mRNA level decreased by more than 30% (p < 0.001) at the concentration of 10 mM and by 50% at 20 mM (p < 0.001). The authors, however, cautioned that the effect might have been dependent on LiCl presence during the early stages of viral replication (first 9 hours) and the increase of tumor necrosis factor-α, which was greater following LiCl alone than induced by the virus ^2^. *In vitro* studies of another porcine coronavirus causing transmissible gastroenteritis indicated that LiCl (5–25 mM) acts on both early and late stages of infection and inhibits apoptosis ^3^. Both virus titer reduction and cell survival at 70–90% were achieved with LiCl at 25 mM (10–50% at 5 mM). The same research group from Harbin in China reported earlier that LiCl (investigated at 5–50 mM) reduced the cytopathic effect of the avian infectious bronchitis virus (also a coronavirus) in primary chicken embryo kidney cells ^4^. The results suggest that the dose of 5 mM was beneficial (20% inhibition) when applied one hour after infection, but not 8 hours post infection. In Vero cells, African green monkey kidney-derived epithelial cells, and immortalized chicken embryo fibroblasts LiCl suppressed the avian coronavirus infectious bronchitis. Relative virus titers in both cell lines were reduced by at least 45% at 5 mM and 70–90% at 10 mM. Viral mRNA concentration decreased 20 times in both cell types cultured with 5 mM LiCl. Overall, the antiviral activity of lithium was ascribed to a cellular effect ^5^. One study was identified outside the main search reports on the activity of high LiCl concentrations (10–60 mM) against porcine deltacoronavirus: at 10 mM 50% relative mRNA reduction was found with no accompanying effect on the viral titer ^6^.

## Discussion

The available evidence comes only from studies of cell cultures and indicates that lithium effectively inhibits coronaviral infections when administered at concentrations that are toxic to humans.

### Putative molecular mechanisms

The major putative molecular mechanisms of antiviral activity and reduced apoptosis is the inhibition of glycogen synthase kinase 3-beta (GSK-3β) ^7, 8^. However, lithium also inhibits GSK-3α, inositol monophosphatases, and may indirectly act via the electrolyte balance.

PEDV requires the PI3K/Akt/GSK-3α/β pathway, which can be targeted at GSK-3β by lithium ^9^. Curiously, GSK-3β is required for template switching, a process seemingly indispensable for the production of coronaviral genomic RNA. The inhibition of GSK-3β prevents longer viral subgenomic mRNAs and the genomic RNA from being synthesized ^10^. Their production would require GSK-3β-dependent phosphorylation of the viral nucleocapsid and subsequent recruitment of helicase DDX1.

Chloroquine (hydroxychloroquine) – which is thought to be effective in COVID-19 ^11^ – was shown to inhibit GSK-3β and potentiate GSK-3β inhibition caused by lithium. This indicates that mechanistic studies could investigate not only 0.5–1.2 mM lithium, but lithium with chloroquine as well. This also brings zinc to the spotlight since zinc inhibits GSK-3β at micromolar concentrations ^12^.

### Known antiviral activity in humans

There is some evidence that lithium may affect the course of viral diseases in humans. In a retrospective cohort study of patients with affective disorders a decrease in the rate of recurrent labial herpes was found in the lithium group (n = 177, p < 0.001) but not in the alternative treatment group (n = 59, p = 0.53) ^13^. In research previously conducted by Prof. J. Rybakowski at our hospital, lithium prevented labial herpes recurrence in thirteen out of 28 eligible psychiatric patients. Lithium also seemed to bring improvement in a proof-of-concept randomized double-blind placebo-controlled trial involving eleven healthy adults with recurrent HSV infections ^14^ and in a randomized study of ten women with genital herpes conducted by the same research group.

### Other evidence for antiviral activity

LiCl was shown to dose-dependently inhibit reovirus (10–60 mM) ^15^ and food-and-mouth disease virus (10–40 mM) ^16^. At 5 mM concentration LiCl reduced the replication of avian leukosis virus subgroup J in chicken embryo fibroblast cells ^17^. Yet, lithium at 50 µM concentration (12–20 times smaller than usually maintained in bipolar disorder) significantly reduced hepatitis C virus copy number (P = 0.0002) in supernatant from Huh7.5 cell culture ^18^. The latter study gives hope that lithium may indeed be efficient at clinically relevant levels.

### Safety and limitations

Lithium carbonate is an orphan drug widely used in the treatment of bipolar disorder. Its safety, when used correctly, is excellent ^19^. The main concern in the setting of an infectious disease unit would be the potential for interactions with other medication, possibly leading to the elevation of lithium levels and acute toxicity, mostly renal. This may be prevented by monitoring serum lithium concentrations. To our best knowledge, no interactions between lithium carbonate and ribavirin, lopinavir or ritonavir exist. A randomized study in tenofovir-treated patients with HIV revealed that 24-week addition of lithium at target serum concentrations of 0.6–1.0 mmol/L was not associated with nephrotoxicity ^20^.

Lithium concentration may be, on the other hand, increased by loop or thiazide diuretics, angiotensin-converting enzyme inhibitors, and non-steroid anti-inflammatory drugs. It is also is not clear if the use of lithium would be safe in acute disease accompanied by dehydration and unstable electrolyte levels. Cardiotoxicity of lithium may occur not only with concentrations larger than 1.5 mmol/L, but also when levels of the ion rapidly change ^21^. Although QTc prolongation is absent in most patients receiving lithium, QT dispersion ratio may increase; longer QT was also described in some cases. Concurrent use of lithium with chloroquine would need to be especially cautious in patients with QT prolongation.

In the light of the reviewed data lithium appears as a possible candidate for therapy of COVID-19. We propose mechanistic investigation of the influence of lithium (0.5–1 mM) – alone and with chloroquine or other drugs – on the SARS-CoV-2 infection.

## Data availability

### Underlying data

All data underlying the results are available as part of the article and no additional source data are required.

### Reporting guidelines

Zenodo: PRISMA ScR checklist for ‘Is lithium a potential treatment for the novel Wuhan (2019-nCoV) coronavirus? A scoping review’. https://doi.org/10.5281/zenodo.3637574 ^22^.

The adapted reporting guidelines checklist is available under the terms of the Creative Commons Attribution 4.0 International license (CC-BY 4.0).
