# Supplementary material for: Lithium and coronaviral infections. A scoping review
Source: F1000Res. 2020 Apr 3;9:93. Originally published 2020 Feb 7. [Version 2] doi: 10.12688/f1000research.22299.2 (PMC7255895; doi:10.12688/f1000research.22299.2)
Supplement: Peer review history

# Peer review: Reviewer response for version 2

**DOI:** 10.5256/f1000research.25289.r63733

**Published:** 2020-07-20

**Authors:** De-Maw Chuang

**Affiliations:** 1 Intramural Research Program, National Institute of Mental Health, National Institutes of Health, Bethesda, MD, USA

In the latest version of this article, the authors have adequately responded to the points raised by previous reviewers and critically evaluated each of these cell culture studies to assess the treatment potential of lithium for COVID-19 infections as well as the limitation and safety issue of this drug in clinic utility. This article is highly meaningful and could pave the way for using lithium to intervene in COVID-19 infections. If space is allowed, a few relatively minor points below are suggested to further enhance the paper:

1. Lithium is a mysteries and powerful drug with the potential to be used in diseases beyond bipolar disorder. Among lithium’s actions, it seems appropriate to bring up the neuroprotective effects of this drug, which has been established for more than 20 years ago since the first publication in this area by Nonaka and colleagues (Nonaka *et al*, PNAS, 95: 2642-2647, 1998) ^1^. At present, lithium has been found by various laboratories to exhibit robust anti-apoptotic, anti-inflammatory and neurotrophic properties, in preclinical settings of a large number of neurodegenerative, neurological and neuropsychiatric diseases (for review, Chiu *et al.*, Phar Review, 65:105-142, 2013) ^2^. This information is relevant to the current review, given that COVID-19 infections were accompanied by the detection of the virus in the patients' brain and cerebrospinal fluid, and could enhance the occurrence of brain disorders such as stroke, depression, encephalitis, seizures, and loss of smelling sensation (e.g., Wu et al., Brain Behav Immun,  Advance online, March 30, 2020) ^3^.  
2. Although supra-therapeutic concentrations of lithium were needed to suppress COVID-19 infection in vitro, this piece of information does not necessarily indicate that toxic doses of this drug are needed to produce ant-coronavirus effects in vivo. In the case of neuroprotective studies of lithium, lower doses of lithium with longer-term treatment were usually sufficient in primary neuronal cultures, while higher lithium doses with shorter-team treatment were often seen in cell- lines studies. In contrast to in vitro studies, clinically relevant doses of lithium were able to produce neuroprotective effects in preclinical animal models of brain disorders, such as stroke, Alzheimer’s and Huntington's diseases, among others (for review, Chiu and Chuang, Pharm Thera 128: 281-304, 2010; Chiu *et al.*, Pharm Rev 65:105-142, 2013) ^4^^,^^2^. 
3. Since there are indications that early treatment with lithium after infections was more effective than delayed treatment against the COVID-19 attacks, the authors may also want to go forward and propose that lithium pretreatment could be used as a prophylactic strategy during the COVID pandemic. As pointed out, the anti-COVID-19 effects of lithium most likely involve GSK3 inhibition, it will be more helpful and informative to mention that lithium can inhibit GSK3 directly by replacing magnesium at the active site or indirectly by enhancing serine phosphorylation of this kinase to reduce its enzymatic activities (for review, Chiu and Chuang, Pharm Thera, 128: 281-304, 2010) ^4^.
4. In the subsection of "Other Evidence for Antiviral Activity" of the Discussion, it is suggested to include statements such as: "Notably, non-toxic concentrations of lithium were also reported to inhibit the replication of HIV via a GSK3-Wnt/β-catenin-dependent mechanism (Kumar *et al.*, J Virol 82: 2813-2820, 2008) ^5^. Additionally, lithium pretreatment protects the hippocampus of mice from HIV-gp120-induced toxicity (Everall *et al.*, Mol Cell Neurosci 21: 493-501, 2002) ^6^."
5. The rationale of using lithium in combination with chloroquine to control COVID-19 infection is weak and unclear, in light of increasing evidence speaking against the effectiveness of chloroquine. At least, some statements have to be made to indicate that the efficacy of chloroquine in COVID patients is debatable and not yet well accepted.

Is the work clearly and accurately presented and does it cite the current literature?

Yes

If applicable, is the statistical analysis and its interpretation appropriate?

Yes

Are all the source data underlying the results available to ensure full reproducibility?

No source data required

Is the study design appropriate and is the work technically sound?

Yes

Are the conclusions drawn adequately supported by the results?

Yes

Are sufficient details of methods and analysis provided to allow replication by others?

Yes

Reviewer Expertise:

Neurobiology of lithium.

I confirm that I have read this submission and believe that I have an appropriate level of expertise to confirm that it is of an acceptable scientific standard.

## References

[1] : Chronic lithium treatment robustly protects neurons in the central nervous system against excitotoxicity by inhibiting N-methyl-D-aspartate receptor-mediated calcium influx. *Proc Natl Acad Sci U S A*.1998;95(5) : 10.1073/pnas.95.5.2642 2642-7 10.1073/pnas.95.5.2642 9482940PMC19446

[2] : Therapeutic potential of mood stabilizers lithium and valproic acid: beyond bipolar disorder. *Pharmacol Rev*.2013;65(1) : 10.1124/pr.111.005512 105-42 10.1124/pr.111.005512 23300133PMC3565922

[3] : Nervous system involvement after infection with COVID-19 and other coronaviruses. *Brain Behav Immun*.2020; 10.1016/j.bbi.2020.03.031 10.1016/j.bbi.2020.03.031 32240762PMC7146689

[4] : Molecular actions and therapeutic potential of lithium in preclinical and clinical studies of CNS disorders. *Pharmacol Ther*.2010;128(2) : 10.1016/j.pharmthera.2010.07.006 281-304 10.1016/j.pharmthera.2010.07.006 20705090PMC3167234

[5] : Active beta-catenin signaling is an inhibitory pathway for human immunodeficiency virus replication in peripheral blood mononuclear cells. *J Virol*.2008;82(6) : 10.1128/JVI.02498-07 2813-20 10.1128/JVI.02498-07 18199649PMC2258975

[6] : Lithium ameliorates HIV-gp120-mediated neurotoxicity. *Mol Cell Neurosci*.2002;21(3) : 10.1006/mcne.2002.1196 493-501 10.1006/mcne.2002.1196 12498789

# Peer review: Reviewer response for version 2

**DOI:** 10.5256/f1000research.25289.r62010

**Published:** 2020-05-27

**Authors:** Fangqiang Wei, Weier Wang

**Affiliations:** 1 Department of Hepatobiliary and Pancreatic Surgery, Zhejiang Provincial People's Hospital, Hangzhou Medical College, Hangzhou, China; 2 Second Clinical Medical College, Zhejiang Chinese Medical University, Hangzhou, Zhejiang, China

The rapid spread of COVID-2019 around the world has caused more than 5 million infections and over 300,000 deaths, which undoubtedly has a huge impact on the global economy and people's health. It is necessary to explore the potential treatment of the disease.

 The two authors described the effects of different concentrations of lithium on coronavirus infections and discussed potential mechanisms based on existing literature. Although the current reports are mostly cell studies, it seems that lithium plays a role in inhibiting coronavirus infection. In the revised version of the paper, the authors state that lithium appears as a possible candidate for therapy of COVID-19. And they also made attempt to investigate the influence of lithium (0.5–1 mM) – alone and with chloroquine or other drugs – on the SARS-CoV-2 infection. Besides, they emphasized the need to monitor blood lithium concentrations, which made their conclusions more rigorous.

 In summary, the prediction of the potential role of lithium in the treatment or prevention of COVID-2019 in this paper is reasonable to some extent. However, so far, there have been no in vivo studies and other relevant conclusive evidence to confirm this hypothesis. It may still be necessary to carry out research on related aspects in the future. Although lithium may not soon be used in the epidemic of COVID-19, it may have a certain role in the next similar outbreak.

 Overall a well-revised manuscript with interesting results. I recommend indexing.

Is the work clearly and accurately presented and does it cite the current literature?

Yes

If applicable, is the statistical analysis and its interpretation appropriate?

Not applicable

Are all the source data underlying the results available to ensure full reproducibility?

Yes

Is the study design appropriate and is the work technically sound?

Partly

Are the conclusions drawn adequately supported by the results?

Partly

Are sufficient details of methods and analysis provided to allow replication by others?

Yes

Reviewer Expertise:

NA

We confirm that we have read this submission and believe that we have an appropriate level of expertise to confirm that it is of an acceptable scientific standard.

# Peer review: Reviewer response for version 2

**DOI:** 10.5256/f1000research.25289.r63730

**Published:** 2020-05-26

**Authors:** Rodrigo Machado-Vieira

**Affiliations:** 1 Department of Psychiatry and Behavioral Sciences, McGovern Medical School, University of Texas Science Center at Houston, Houston, TX, USA

The authors provided a systematic approach to identify six *in vitro *studies reporting on the influence of lithium on coronaviral infections. The discussion on the potential role of lithium is hypothetical and the proposed key target does not seem relevant in the context of COVID-19.

 Some comments: Ref 2: At 5 mM LiCl reduced viral RNA levels by 30% (p < 0.001), this level is commonly lethal to humans (and when not fatal, may be associated with respiratory failure, a hallmark in COVID-19. In the same study, therapeutic levels did not work the same way, thus it seems that its antiviral effects are related to lithium’s overall toxicity; also considering the higher levels (10 and 20mM) showed a linear decrease in viral mRNA. Similar effects may be applied to references 3 and 4, which also describe supra-therapeutic levels of lithium as potential anti-viral, which is hard to translate into potential therapeutics. In line with the presented findings, lithium showed to ameliorate HIV-gp120-mediated neurotoxicity. GSk3-B is a widespread target for lithium effects and based its wide distribution in several organs and having several other subunits targeted as well as GSK-3B inhibitors failed in early studies with most of the related agents and did not mimic any clinical effects of lithium. Thus, the enthusiasm of its potential utility as a treatment target in drug development (e.g. mood disorders) has been significantly diminished in the last few years. Also, the risk or severity of QTc prolongation can be increased when chloroquine is combined with lithium, and Gsk-3 is not a target directly associated with COVID-19 in any published study so far. Effects of lithium on HIV seem much more beneficial than associated with worse outcomes and this potential effect seems to be relevant in the context.

 Description of safety and limitations are generic in terms of lithium effects, except for the QT prolongation in combination with chloroquine, do not highlight the specific aspects related to the author’s hypothesis, such as cell proliferation. Evidence for the antiviral effects of lithium in prospective clinical studies remains to be presented. Also, the suggestion of a similar role for lithium and zinc in this context of COVID-19 should be further explained in terms of molecular and ionic convergent effects. Lithium chloride does not have a direct virucidal effect on coronavirus infectious bronchitis virus infection (Harrison SM, *et al.*, ref 5).

 Before starting using lithium in COVID-19, a potential recommendation is a comprehensive evaluation of COVID-19 outcomes in subjects with Bipolar disorder on vs. off lithium treatment.

Is the work clearly and accurately presented and does it cite the current literature?

Yes

If applicable, is the statistical analysis and its interpretation appropriate?

Not applicable

Are all the source data underlying the results available to ensure full reproducibility?

Yes

Is the study design appropriate and is the work technically sound?

Partly

Are the conclusions drawn adequately supported by the results?

Partly

Are sufficient details of methods and analysis provided to allow replication by others?

Partly

Reviewer Expertise:

Lithium, biomarkers, clinical trials

I confirm that I have read this submission and believe that I have an appropriate level of expertise to confirm that it is of an acceptable scientific standard.

## References

[1] : Lithium, Stress, and Resilience in Bipolar Disorder: Deciphering this key homeostatic synaptic plasticity regulator. *J Affect Disord*.233: 10.1016/j.jad.2017.12.026 92-99 10.1016/j.jad.2017.12.026 29310970

[2] : The role of lithium in the treatment of bipolar disorder: convergent evidence for neurotrophic effects as a unifying hypothesis. *Bipolar Disord*.2009;11 Suppl 2: 10.1111/j.1399-5618.2009.00714.x 92-109 10.1111/j.1399-5618.2009.00714.x 19538689PMC2800957

# Peer review: Reviewer response for version 2

**DOI:** 10.5256/f1000research.25289.r62011

**Published:** 2020-04-09

**Authors:** Jean-Martin Beaulieu

**Affiliations:** 1 Department of Pharmacology & Toxicology, University of Toronto, Toronto, ON, Canada

The possibility that lithium may be useful for corona virus infections remains hypothetical. This is now acknowledged and the article presents the limitations of this approach. A possible supporting study would be to compare disease severity in lithium treated vs non-lithium treated subjects in patients receiving these drugs in the context of the Covid 19 epidemic. Since several psychoactive drugs (e.g. antipsychotic) also affect putative molecular targets of lithium (e.g.GSK3alpha and beta) ^1^, ^2^, groups treated with these drugs would be interesting to compare to lithium.   However, several other factors affecting people with psychiatric illness therapies may also affect the impact of Covid19 or other corona viruses on these populations. This is obviously beyond the scope of this review/hypothesis article. ​

Is the work clearly and accurately presented and does it cite the current literature?

Yes

If applicable, is the statistical analysis and its interpretation appropriate?

Not applicable

Are all the source data underlying the results available to ensure full reproducibility?

Yes

Is the study design appropriate and is the work technically sound?

Partly

Are the conclusions drawn adequately supported by the results?

No

Are sufficient details of methods and analysis provided to allow replication by others?

Yes

Reviewer Expertise:

Gsk3 signaling

I confirm that I have read this submission and believe that I have an appropriate level of expertise to confirm that it is of an acceptable scientific standard.

## References

[1] : A role for Akt and glycogen synthase kinase-3 as integrators of dopamine and serotonin neurotransmission in mental health. *J Psychiatry Neurosci*.2012;37(1) : 10.1503/jpn.110011 7-16 10.1503/jpn.110011 21711983PMC3244494

[2] : Akt/GSK3 signaling in the action of psychotropic drugs. *Annu Rev Pharmacol Toxicol*.2009;49: 10.1146/annurev.pharmtox.011008.145634 327-47 10.1146/annurev.pharmtox.011008.145634 18928402

# Peer review: Reviewer response for version 1

**DOI:** 10.5256/f1000research.24598.r59736

**Published:** 2020-02-21

**Authors:** Jean-Martin Beaulieu

**Affiliations:** 1 Department of Pharmacology & Toxicology, University of Toronto, Toronto, ON, Canada

The authors identified five previous studies reporting an effect of lithium (mostly LiCl) in corona virus in cellular systems. This is obviously a very timely question. All studies point toward beneficial effects of lithium and thus underscore the possible beneficial effect of targeting lithium sensitive biochemical pathways, namely GSK3 mediated signaling for corona virus treatment or prophylaxis.

 As a technical issue lithium not only targets GSK3b but also GSK3-alpha and inositol monophosphatases. So the emphasis on GSK3-beta may be a bit premature. 

 A more important issue is that none of the studies shown an effect of lithium at a 1-1.5mM concentrations. Effects are reported at Li+ concentrations that are 5mM or higher. These concentrations are not toxic for cells in culture. However, in humans, serum lithium concentration above 1.5-2.0mM (or mEq, which stands for the mM concentration of the lithium ion) are considered toxic (Haussmann *et al.*, 2015 ^1^).

 The prescription of lithium in the context of the current epidemic thus appears not to be supportable by the findings. The cure may kill the patients. 

 More detailed studies using lithium in animal models at tolerable concentrations would thus be needed. 

 Unfortunately these limitations are not addressed in the manuscript.

Is the work clearly and accurately presented and does it cite the current literature?

Yes

If applicable, is the statistical analysis and its interpretation appropriate?

Not applicable

Are all the source data underlying the results available to ensure full reproducibility?

Yes

Is the study design appropriate and is the work technically sound?

Partly

Are the conclusions drawn adequately supported by the results?

No

Are sufficient details of methods and analysis provided to allow replication by others?

Yes

Reviewer Expertise:

Gsk3 signaling

I confirm that I have read this submission and believe that I have an appropriate level of expertise to state that I do not consider it to be of an acceptable scientific standard, for reasons outlined above.

## References

[1] : Treatment of lithium intoxication: facing the need for evidence. *Int J Bipolar Disord*.2015;3(1) : 10.1186/s40345-015-0040-2 23 10.1186/s40345-015-0040-2 26493348PMC4615994

# Response

**Published:** 2020-03-17

**Authors:** Jan Nowak

**Affiliations:** Poznan University of Medical Sciences, Poland

Poznań, March 16 ^th^, 2020

Dear Prof. Beaulieu,

We are grateful for the comments that you have provided. They helped to improve our manuscript. Please find our responses below.

Sincerely yours,

Jan Nowak and Jarosław Walkowiak

**The authors identified five previous studies reporting an effect of lithium (mostly LiCl) in corona virus in cellular systems. This is obviously a very timely question. All studies point toward beneficial effects of lithium and thus underscore the possible beneficial effect of targeting lithium sensitive biochemical pathways, namely GSK3 mediated signaling for corona virus treatment or prophylaxis.**

**As a technical issue lithium not only targets GSK3b but also GSK3-alpha and inositol monophosphatases. So the emphasis on GSK3-beta may be a bit premature.**

We thank the reviewer for this comment. This is now referred to early in the discussion:

- *“The major putative molecular mechanisms of antiviral activity and reduced apoptosis is the inhibition of glycogen synthase kinase 3-beta (GSK-3β) ^7,8^. However, lithium also inhibits GSK-3α, inositol monophosphatases, and may indirectly act via the electrolyte balance.”*

**A more important issue is that none of the studies shown an effect of lithium at a 1-1.5mM concentrations. Effects are reported at Li+ concentrations that are 5mM or higher. These concentrations are not toxic for cells in culture. However, in humans, serum lithium concentration above 1.5-2.0mM (or mEq, which stands for the mM concentration of the lithium ion) are considered toxic (Haussmann et al., 20151).**

This important comment has triggered a number of major changes in the manuscript. In order not to mislead the readers we propose a new title, which is more neutral:

- *“Lithium and coronaviral infections. A scoping review.”*

The conclusion of the abstract was changed:

- *“We propose mechanistic investigation of the influence of lithium – alone and with chloroquine – on the SARS-CoV-2 infection.”*

The conclusion at the end of the discuss was altered:

- *“In the light of the reviewed data lithium appears as a possible candidate for therapy of COVID-2019. We propose mechanistic investigation of the influence of lithium (0.5-1 mM) – alone and with chloroquine or other drugs – on the SARS-CoV-2 infection.”*

Throughout the results section we now report and comment on LiCl concentrations, which are clearly above the levels, which are safe for humans. Examples:

- *“In Vero cells, lithium chloride (investigated at 1–15 mM) was shown to be dose-dependently effective in suppressing infection with the porcine epidemic diarrhea virus (PEDV), a member of the Coronaviridae family ^1^.“*
- *“Yet, LiCl at 1 mM (safe in patients) was not effective. At 5 mM LiCl reduced viral RNA levels by 30% (p < 0.001).”*
- *“The relative viral mRNA level decreased by more than 30% (p < 0.001) at the concentration of 10 mM and by 50% at 20 mM (p < 0.001).”*
- *“Both virus titer reduction and cell survival at 70–90% were achieved with LiCl at 25 mM (10–50% at 5 mM).”*
- *“The results suggest that the dose of 5 mM was beneficial (20% inhibition) when applied one hour after infection, but not 8 hours post infection.”*
- *“Relative virus titers in both cell lines were reduced by at least 45% at 5 mM and 70–90% at 10 mM. Viral mRNA concentration decreased 20 times in both cell types cultured with 5 mM LiCl.”*
- *“One study was identified outside the main search reports on the activity of high LiCl concentrations (10-60 mM) against porcine deltacoronavirus: at 10 mM 50% relative mRNA reduction was found with no accompanying effect on the viral titer ^6^.”*

Crucially, the discussion now opens with:

- “The available evidence comes only from studies of cell cultures and indicates that lithium effectively inhibits coronaviral infections when administered at concentrations that are toxic to humans.”

**The prescription of lithium in the context of the current epidemic thus appears not to be supportable by the findings. The cure may kill the patients.**

As cited above, the discussion now starts by stating:

- *“The available evidence comes only from studies of cell cultures and indicates that lithium effectively inhibits coronaviral infections when administered at concentrations that are toxic to humans.”*

However, hypothesizing that lithium could be useful in treating viral infections is now supported by some other evidence:

- *“There is some evidence that lithium may affect the course of viral diseases in humans. In a retrospective cohort study of patients with affective disorders a decrease in the rate of recurrent labial herpes was found in the lithium group (n = 177, p < 0.001) but not in the alternative treatment group (n = 59, p = 0.53) ^13^. In research previously conducted by Prof. J. Rybakowski at our hospital, lithium prevented labial herpes recurrence in thirteen out of 28 eligible psychiatric patients. Lithium also seemed to bring improvement in a proof-of-concept randomized double-blind placebo-controlled trial involving eleven healthy adults with recurrent HSV infections ^14^ and in a randomized study of ten women with genital herpes conducted by the same research group from Philadelphia.”*

Therefore it seems that in some instances lithium exhibits antiviral activity at concentrations, which are safe and maintained long-term (for years) in patients with affective disorders.

Additionally, the discussion of lithium safety is now broader.

- *“Lithium concentration may be, on the other hand, increased by loop or thiazide diuretics, angiotensin-converting enzyme inhibitors, and non-steroid anti-inflammatory drugs. It is also is not clear if the use of lithium would be safe in acute disease accompanied by dehydration and unstable electrolyte levels. Cardiotoxicity of lithium may occur not only with concentrations larger than 1.5 mmol/L, but also when levels of the ion rapidly change ^21^. Although QTc prolongation is absent in most patients receiving lithium, QT dispersion ratio may increase; longer QT was also described in some cases. Concurrent use of lithium with chloroquine would need to be especially cautious in patients with QT prolongation.”*

**More detailed studies using lithium in animal models at tolerable concentrations would thus be needed.**

As we mentioned, this has been adapted to be the conclusion of our study.

- *“In the light of the reviewed data lithium appears as a possible candidate for therapy of COVID-2019. We propose mechanistic investigation of the influence of lithium (0.5-1 mM) – alone and with chloroquine or other drugs – on the SARS-CoV-2 infection.”*

**Unfortunately these limitations are not addressed in the manuscript.**

We thank for the critique, which has helped to transform our manuscript.

The references were updated

1 

Li HJ, Gao DS, Li YT, et al.: Antiviral effect of lithium chloride on porcine epidemic diarrhea virus *in vitro*. *Res Vet Sci.* 2018;118:288–94. 29547727 10.1016/j.rvsc.2018.03.002

2 

Cui J, Xie J, Gao M, et al.: Inhibitory effects of lithium chloride on replication of type II porcine reproductive and respiratory syndrome virus *in vitro*. *Antivir Ther.* 2015;20(6):565–72. 25560301 10.3851/IMP2924

3 

Ren X, Meng F, Yin J, et al.: Action mechanisms of lithium chloride on cell infection by transmissible gastroenteritis coronavirus. *PLoS One.* 2011;6(5):e18669. 21573100 10.1371/journal.pone.0018669 3089605

4 

Li J, Yin J, Sui X, et al.: Comparative analysis of the effect of glycyrrhizin diammonium and lithium chloride on infectious bronchitis virus infection *in vitro*. *Avian Pathol.* 2009;38(3):215–21. 19468938 10.1080/03079450902912184

5 

Harrison SM, Tarpey I, Rothwell L, et al.: Lithium chloride inhibits the coronavirus infectious bronchitis virus in cell culture. *Avian Pathol.* 2007;36(2):109–14. 17479370 10.1080/03079450601156083

6 

 Zhai X, Wang S, Zhu M, He W, Pan Z, Su S. Antiviral Effect of Lithium Chloride and Diammonium Glycyrrhizinate on Porcine Deltacoronavirus In Vitro. Pathogens. 2019 Sep;8(3):144.

7

Zaheer A, Knight S, Zaheer A, et al.: Glia maturation factor overexpression in neuroblastoma cells activates glycogen synthase kinase-3beta and caspase-3. *Brain Res.* 2008;1190:206–14. 18054898 10.1016/j.brainres.2007.11.011 2343001

8 

Choi SE, Kang Y, Jang HJ, et al.: Involvement of glycogen synthase kinase-3beta in palmitate-induced human umbilical vein endothelial cell apoptosis. *J Vasc Res.* 2007;44(5):365–74. 17483602 10.1159/000102321

9 

Kong N, Wu Y, Meng Q, et al.: Suppression of Virulent Porcine Epidemic Diarrhea Virus Proliferation by the PI3K/Akt/GSK-3α/β Pathway. *PLoS One.* 2016;11(8):e0161508. 27560518 10.1371/journal.pone.0161508 4999130

10 

Wu CH, Chen PJ, Yeh SH: Nucleocapsid phosphorylation and RNA helicase DDX1 recruitment enables coronavirus transition from discontinuous to continuous transcription. *Cell Host Microbe.* 2014;16(4):462–72. 25299332 10.1016/j.chom.2014.09.009

11

Multicenter collaboration group of Department of Science and Technology of Guangdong Province and Health Commission of Guangdong Province for chloroquine in the treatment of novel coronavirus pneumonia. [Expert consensus on chloroquine phosphate for the treatment of novel coronavirus pneumonia]. Zhonghua Jie He He Hu Xi Za Zhi. 2020 Mar 12;43(3):185–8.

12

Eldar-Finkelman H, Martinez A. GSK-3 Inhibitors: Preclinical and Clinical Focus on CNS. Front Mol Neurosci. 2011;4:32.

13

Amsterdam JD, Maislin G, Rybakowski J. A possible antiviral action of lithium carbonate in herpes simplex virus infections. Biol Psychiatry. 1990 Feb 15;27(4):447–53.

14

Amsterdam JD, Maislin G, Hooper MB. Suppression of herpes simplex virus infections with oral lithium carbonate--a possible antiviral activity. Pharmacotherapy. 1996 Dec;16(6):1070–5.

15

Chen Y, Kong D, Cai G, Jiang Z, Jiao Y, Shi Y, et al. Novel antiviral effect of lithium chloride on mammalian orthoreoviruses in vitro. Microb Pathog. 2016 Apr;93:152–7.

16

Zhao F-R, Xie Y-L, Liu Z-Z, Shao J-J, Li S-F, Zhang Y-G, et al. Lithium chloride inhibits early stages of foot-and-mouth disease virus (FMDV) replication in vitro. J Med Virol. 2017;89(11):2041–6.

17

Qian K, Cheng X, Zhang D, Shao H, Yao Y, Nair V, et al. Antiviral effect of lithium chloride on replication of avian leukosis virus subgroup J in cell culture. Arch Virol. 2018 Apr 1;163(4):987–95.

18

Sarhan MA, Abdel-Hakeem MS, Mason AL, Tyrrell DL, Houghton M. Glycogen synthase kinase 3β inhibitors prevent hepatitis C virus release/assembly through perturbation of lipid metabolism. Sci Rep [Internet]. 2017 May 31 [cited 2020 Mar 14];7. Available from: https://www.ncbi.nlm.nih.gov/pmc/articles/PMC5451429/

19 

Rybakowski JK: Challenging the Negative Perception of Lithium and Optimizing Its Long-Term Administration. *Front Mol Neurosci.* 2018;11:349. 30333722 10.3389/fnmol.2018.00349 6175994

20

Decloedt EH, Lesosky M, Maartens G, Joska JA. Renal safety of lithium in HIV-infected patients established on tenofovir disoproxil fumarate containing antiretroviral therapy: analysis from a randomized placebo-controlled trial. AIDS Res Ther. 2017 Feb 4;14(1):6.

21

Mehta N, Vannozzi R. Lithium-induced electrocardiographic changes: A complete review. Clinical Cardiology. 2017;40(12):1363–7.

22 

Nowak JK, Walkowiak J: PRISMA Scoping Review checklist for Nowak and Walkowiak 2020. *Zenodo.* 2020. http://www.doi.org/10.5281/zenodo.3637575

# Peer review: Reviewer response for version 1

**DOI:** 10.5256/f1000research.24598.r59741

**Published:** 2020-02-12

**Authors:** Fangqiang Wei, Weier Wang

**Affiliations:** 1 Department of Hepatobiliary and Pancreatic Surgery, Zhejiang Provincial People's Hospital, Hangzhou Medical College, Hangzhou, China; 2 Second Clinical Medical College, Zhejiang Chinese Medical University, Hangzhou, Zhejiang, China

The wide spread of infection of 2019-nCoV has arouse an international concern since its original outbreak  in Wuhan, China. Scientists and health workers around the world are currently working together to wipe out the virus and the novel coronavirus pneumonia (NCP), which has killed more than a thousand lives, by far, worldwide.

 With the current epidemic being so severe, it is necessary and urgent to make potentially reasonable recommendations for the treatment or prevention for 2019-nCoV or NCP. The two authors clearly proposed that lithium might be a potential treatment or prophylaxis for 2019-nCoV or NCP based on a summary of existing literature that reported the *in vitro* effects of lithium on coronaviral infections and discussed potential mechanisms, which sound reasonable to some extent, but still not rigorous.

 Specifically, there are few related studies available and only *in vitro* data have been reported. The authors may need more related studies and solid evidence to support their hypothesis to make it more scientific and rigorous. As reported, lithium can be toxic due to its side effects, mainly thyroid, renal, and cognitive disturbances. Readers may wish to see more clinical information of lithium in treating viral infection cases, if not available, or in treating other diseases.

 In terms of discussion, the authors reviewed some existing literature and suggested a potential mechanism of reduced apoptosis by lithium, the glycogen synthase kinase 3-beta (GSK-3β) inhibitor. The possibility that targeting at GSK-3β by lithium may potentially affect the coronavirus is an interesting topic. However, direct *in vitro* evidence is lacking regarding 2019-nCoV or related coronaviruses including severe acute respiratory syndrome (SARS) and Middle East respiratory syndrome (MERS) coronaviruses.

 Moreover, relevant literature is still needed although the authors state that there is no interaction between lithium carbonate and ribavirin, lopinavir, or ritonavir exist. Another aspect worth noting is that the authors indicate that monitoring serum lithium concentration can be helpful in preventing side effects of lithium, however it should be emphasized that the *in vivo* relationship between the effective dose and toxic dose of lithium is still unclear, with some studies reporting a dose-dependent manner of the inhibitory effect of lithium *in vitro*. Thus, it warrants more data, both *in vitro* and *in vivo*, to clarify this issue.

 Collectively, this study proposes a potential role of lithium in treating or preventing 2019-nCoV or NCP with some possible mechanisms. However, by far, solid evidence is lacking to validate this hypothesis. The time of developing lithium orotate for clinical use, even in emergency, is not yet.

Is the work clearly and accurately presented and does it cite the current literature?

Yes

If applicable, is the statistical analysis and its interpretation appropriate?

Not applicable

Are all the source data underlying the results available to ensure full reproducibility?

Yes

Is the study design appropriate and is the work technically sound?

Partly

Are the conclusions drawn adequately supported by the results?

Partly

Are sufficient details of methods and analysis provided to allow replication by others?

Yes

Reviewer Expertise:

NA

We confirm that we have read this submission and believe that we have an appropriate level of expertise to confirm that it is of an acceptable scientific standard, however we have significant reservations, as outlined above.

# Response

**Published:** 2020-03-17

**Authors:** Jan Nowak

**Affiliations:** Poznan University of Medical Sciences, Poland

Poznań, March 16 ^th^, 2020

Dear Prof. Wei and Prof. Wang,

We would like to thank you for all the comments. They helped to improve the manuscript. Please find our responses below.

Sincerely yours,

Jan Nowak and Jarosław Walkowiak

**The wide spread of infection of 2019-nCoV has arouse an international concern since its original outbreak  in Wuhan, China. Scientists and health workers around the world are currently working together to wipe out the virus and the novel coronavirus pneumonia (NCP), which has killed more than a thousand lives, by far, worldwide.**

**With the current epidemic being so severe, it is necessary and urgent to make potentially reasonable recommendations for the treatment or prevention for 2019-nCoV or NCP. The two authors clearly proposed that lithium might be a potential treatment or prophylaxis for 2019-nCoV or NCP based on a summary of existing literature that reported the in vitro effects of lithium on coronaviral infections and discussed potential mechanisms, which sound reasonable to some extent, but still not rigorous.**

We appreciate the critique, which has helped to improve our manuscript.

**Specifically, there are few related studies available and only in vitro data have been reported. The authors may need more related studies and solid evidence to support their hypothesis to make it more scientific and rigorous.**

More details are provided in the results. New paragraphs also discuss the antiviral activity in humans and cell cultures challenged with other viruses.

**As reported, lithium can be toxic due to its side effects, mainly thyroid, renal, and cognitive disturbances. Readers may wish to see more clinical information of lithium in treating viral infection cases, if not available, or in treating other diseases.**

Lithium cardiotoxicity is now discussed in more detail.

- *“Lithium concentration may be, on the other hand, increased by loop or thiazide diuretics, angiotensin-converting enzyme inhibitors, and non-steroid anti-inflammatory drugs. It is also is not clear if the use of lithium would be safe in acute disease accompanied by dehydration and unstable electrolyte levels. Cardiotoxicity of lithium may occur not only with concentrations larger than 1.5 mmol/L, but also when levels of the ion rapidly change ^21^. Although QTc prolongation is absent in most patients receiving lithium, QT dispersion ratio may increase; longer QT was also described in some cases. Concurrent use of lithium with chloroquine would need to be especially cautious in patients with QT prolongation.”*

A paragraph on lithium and herpes infections in patients with affective disorders was added. The results of the cited studies are the best evidence for the antiviral activity of lithium that comes from studies conducted in patients.

- *“There is some evidence that lithium may affect the course of viral diseases in humans. In a retrospective cohort study of patients with affective disorders a decrease in the rate of recurrent labial herpes was found in the lithium group (n = 177, p < 0.001) but not in the alternative treatment group (n = 59, p = 0.53) ^13^. In research previously conducted by Prof. J. Rybakowski at our hospital, lithium prevented labial herpes recurrence in thirteen out of 28 eligible psychiatric patients. Lithium also seemed to bring improvement in a proof-of-concept randomized double-blind placebo-controlled trial involving eleven healthy adults with recurrent HSV infections ^14^ and in a randomized study of ten women with genital herpes conducted by the same research group from Philadelphia.”*

More information on the activity of lithium in other viral infections was also provided.

- *“LiCl was shown to dose-dependently inhibit reovirus (10-60 mM) ^15^ and food-and-mouth disease virus (10-40 mM) ^16^. At 5 mM concentration LiCl reduced the replication of avian leukosis virus subgroup J in chicken embryo fibroblast cells ^17^. Yet, lithium at 50 µM concentration (12-20 times smaller than usually maintained in bipolar disorder) significantly reduced hepatitis C virus copy number (P = 0.0002) in supernatant from Huh7.5 cell culture ^18^. The latter study gives hope that lithium may indeed be efficient at clinically relevant levels.”*

** In terms of discussion, the authors reviewed some existing literature and suggested a potential mechanism of reduced apoptosis by lithium, the glycogen synthase kinase 3-beta (GSK-3β** **) inhibitor. The possibility that targeting at GSK-3β** **by lithium may potentially affect the coronavirus is an interesting topic. However, direct in vitro evidence is lacking regarding 2019-nCoV or related coronaviruses including severe acute respiratory syndrome (SARS) and Middle East respiratory syndrome (MERS) coronaviruses.**

Certainly, the evidence is lacking. The discussion is speculative. We have added the following:

- *"The major putative molecular mechanisms of antiviral activity and reduced apoptosis is the inhibition of glycogen synthase kinase 3-beta (GSK-3β) ^7,8^. However, lithium also inhibits GSK-3α, inositol monophosphatases, and may indirectly act via the electrolyte balance."*

And also:

- *“Chloroquine (hydroxychloroquine) – which is thought to be effective in COVID-2019 ^11^ – was shown to inhibit GSK-3β and potentiate GSK-3β inhibition caused by lithium. This indicates that mechanistic studies could investigate not only 0.5-1.2 mM lithium, but lithium with chloroquine as well. This also brings zinc to the spotlight since zinc inhibits GSK-3β at micromolar concentrations ^12^.”*

** Moreover, relevant literature is still needed although the authors state that there is no interaction between lithium carbonate and ribavirin, lopinavir, or ritonavir exist.**

Information on tenofovir is now provided:

- *“A randomized study in tenofovir-treated patients with HIV revealed that 24-week addition of lithium at target serum concentrations of 0.6-1.0 mmol/L was not associated with nephrotoxicity ^20^.”*

**Another aspect worth noting is that the authors indicate that monitoring serum lithium concentration can be helpful in preventing side effects of lithium, however it should be emphasized that the in vivo relationship between the effective dose and toxic dose of lithium is still unclear, with some studies reporting a dose-dependent manner of the inhibitory effect of lithium in vitro. Thus, it warrants more data, both in vitro and in vivo, to clarify this issue.**

Throughout the results section, information on the concentrations of lithium were given and commented on.

Moreover, the discussion opens with:

- *“The available evidence comes only from studies of cell cultures and indicates that lithium effectively inhibits coronaviral infections when administered at concentrations that are toxic to humans.”*

** Collectively, this study proposes a potential role of lithium in treating or preventing 2019-nCoV or NCP with some possible mechanisms. However, by far, solid evidence is lacking to validate this hypothesis. The time of developing lithium orotate for clinical use, even in emergency, is not yet.**

The reference to lithium orotate was removed.

The conclusion was changed:

- *“In the light of the reviewed data lithium appears as a possible candidate for therapy of COVID-2019. We propose mechanistic investigation of the influence of lithium (0.5-1 mM) – alone and with chloroquine or other drugs – on the SARS-CoV-2 infection.”*

We would like the reviewers for their input, which has guided us in improving the text.

Please note that following the remarks of Prof. Beaulieu and that change of the nomenclature (2019-nCoV no longer used) we have proposed a new title, which seems more neutral and therefore more representative of the softened conclusions:

- *“Lithium and coronaviral infections. A scoping review.”*

 

The references were updated

1 

Li HJ, Gao DS, Li YT, et al.: Antiviral effect of lithium chloride on porcine epidemic diarrhea virus *in vitro*. *Res Vet Sci.* 2018;118:288–94. 29547727 10.1016/j.rvsc.2018.03.002

2 

Cui J, Xie J, Gao M, et al.: Inhibitory effects of lithium chloride on replication of type II porcine reproductive and respiratory syndrome virus *in vitro*. *Antivir Ther.* 2015;20(6):565–72. 25560301 10.3851/IMP2924

3 

Ren X, Meng F, Yin J, et al.: Action mechanisms of lithium chloride on cell infection by transmissible gastroenteritis coronavirus. *PLoS One.* 2011;6(5):e18669. 21573100 10.1371/journal.pone.0018669 3089605

4 

Li J, Yin J, Sui X, et al.: Comparative analysis of the effect of glycyrrhizin diammonium and lithium chloride on infectious bronchitis virus infection *in vitro*. *Avian Pathol.* 2009;38(3):215–21. 19468938 10.1080/03079450902912184

5 

Harrison SM, Tarpey I, Rothwell L, et al.: Lithium chloride inhibits the coronavirus infectious bronchitis virus in cell culture. *Avian Pathol.* 2007;36(2):109–14. 17479370 10.1080/03079450601156083

6 

 Zhai X, Wang S, Zhu M, He W, Pan Z, Su S. Antiviral Effect of Lithium Chloride and Diammonium Glycyrrhizinate on Porcine Deltacoronavirus In Vitro. Pathogens. 2019 Sep;8(3):144.

7

Zaheer A, Knight S, Zaheer A, et al.: Glia maturation factor overexpression in neuroblastoma cells activates glycogen synthase kinase-3beta and caspase-3. *Brain Res.* 2008;1190:206–14. 18054898 10.1016/j.brainres.2007.11.011 2343001

8 

Choi SE, Kang Y, Jang HJ, et al.: Involvement of glycogen synthase kinase-3beta in palmitate-induced human umbilical vein endothelial cell apoptosis. *J Vasc Res.* 2007;44(5):365–74. 17483602 10.1159/000102321

9 

Kong N, Wu Y, Meng Q, et al.: Suppression of Virulent Porcine Epidemic Diarrhea Virus Proliferation by the PI3K/Akt/GSK-3α/β Pathway. *PLoS One.* 2016;11(8):e0161508. 27560518 10.1371/journal.pone.0161508 4999130

10 

Wu CH, Chen PJ, Yeh SH: Nucleocapsid phosphorylation and RNA helicase DDX1 recruitment enables coronavirus transition from discontinuous to continuous transcription. *Cell Host Microbe.* 2014;16(4):462–72. 25299332 10.1016/j.chom.2014.09.009

11

Multicenter collaboration group of Department of Science and Technology of Guangdong Province and Health Commission of Guangdong Province for chloroquine in the treatment of novel coronavirus pneumonia. [Expert consensus on chloroquine phosphate for the treatment of novel coronavirus pneumonia]. Zhonghua Jie He He Hu Xi Za Zhi. 2020 Mar 12;43(3):185–8.

12

Eldar-Finkelman H, Martinez A. GSK-3 Inhibitors: Preclinical and Clinical Focus on CNS. Front Mol Neurosci. 2011;4:32.

13

Amsterdam JD, Maislin G, Rybakowski J. A possible antiviral action of lithium carbonate in herpes simplex virus infections. Biol Psychiatry. 1990 Feb 15;27(4):447–53.

14

Amsterdam JD, Maislin G, Hooper MB. Suppression of herpes simplex virus infections with oral lithium carbonate--a possible antiviral activity. Pharmacotherapy. 1996 Dec;16(6):1070–5.

15

Chen Y, Kong D, Cai G, Jiang Z, Jiao Y, Shi Y, et al. Novel antiviral effect of lithium chloride on mammalian orthoreoviruses in vitro. Microb Pathog. 2016 Apr;93:152–7.

16

Zhao F-R, Xie Y-L, Liu Z-Z, Shao J-J, Li S-F, Zhang Y-G, et al. Lithium chloride inhibits early stages of foot-and-mouth disease virus (FMDV) replication in vitro. J Med Virol. 2017;89(11):2041–6.

17

Qian K, Cheng X, Zhang D, Shao H, Yao Y, Nair V, et al. Antiviral effect of lithium chloride on replication of avian leukosis virus subgroup J in cell culture. Arch Virol. 2018 Apr 1;163(4):987–95.

18

Sarhan MA, Abdel-Hakeem MS, Mason AL, Tyrrell DL, Houghton M. Glycogen synthase kinase 3β inhibitors prevent hepatitis C virus release/assembly through perturbation of lipid metabolism. Sci Rep [Internet]. 2017 May 31 [cited 2020 Mar 14];7. Available from: https://www.ncbi.nlm.nih.gov/pmc/articles/PMC5451429/

19 

Rybakowski JK: Challenging the Negative Perception of Lithium and Optimizing Its Long-Term Administration. *Front Mol Neurosci.* 2018;11:349. 30333722 10.3389/fnmol.2018.00349 6175994

20

Decloedt EH, Lesosky M, Maartens G, Joska JA. Renal safety of lithium in HIV-infected patients established on tenofovir disoproxil fumarate containing antiretroviral therapy: analysis from a randomized placebo-controlled trial. AIDS Res Ther. 2017 Feb 4;14(1):6.

21

Mehta N, Vannozzi R. Lithium-induced electrocardiographic changes: A complete review. Clinical Cardiology. 2017;40(12):1363–7.

22 

Nowak JK, Walkowiak J: PRISMA Scoping Review checklist for Nowak and Walkowiak 2020. *Zenodo.* 2020. http://www.doi.org/10.5281/zenodo.3637575
